# Flowers for Hospitals and Schools

**Published:** 1887-01-08

**Authors:** 


### THE EDITOR'S LETTER-BOX.

[Our Correspondents are reminded that prolixity is a great bar to publication, and that brevity of style and conciseness of statement greatly facilitate early publication.]

### FLOWERS FOR HOSPITALS AND SCHOOLS.

A HOSPITAL visitor writes:—"May I beg the favour of a small space in your valuable paper to call the attention of member of flower-missions, and of your wealthy readers who send flowers to hospitals, to the fact that these *sometimes* have a surfeit. What then shall be done with the larger blooms and flowers that have served as decorations in our churches? Let them be sent to the Board schools where some pupil-teacher might, at the breaking-up of the school, stand at the door and distribute his or her treasures to the homeward-bound children; and where is the careworn mother who would not welcome such an adornment to her squalid surroundings? Besides, to children themselves, flowers are a real joy as is proved by an incident witnessed in one of the bye-streets of our city. A little ragged, shoeless boy and girl stood, hand-in-hand before a shop window where bunches of garden flowers were interspersed with currants and raspberries, etc., and all within reach of the passers-by. My sordid thought was that the children were longing for the fruit. Not so. When the boy at last dragged his little sister onward, she turned her head and said in touching tones, 'Pretty Flowers.' How mournful a thought does this bring to our mind!

"And now permit me, in conclusion, to ask the possessors and bestowers of flowers to think of the myriad children in our large towns who never see a flower from year's end to year's end, and not leave them uncared for."

